# Telemedicine: The New Vanguard in Gynecological Care

**DOI:** 10.7759/cureus.88652

**Published:** 2025-07-24

**Authors:** Nilanchali Singh, Saumya Kulshrestha, Nimisha Agrawal, Deepali Garg, Rinchen Zangmo, Sushmitha Somagattu, Akanksha Yadav, K K Roy

**Affiliations:** 1 Department of Obstetrics and Gynaecology, All India Institute of Medical Sciences, New Delhi, New Delhi, IND; 2 Department of Obstetrics and Gynaecology, Luton and Dunstable Hospital United Kingdom, Bedfordshire, GBR

**Keywords:** antenatal care, covid pandemic, maternal-fetal outcomes, patient satisfaction, remote patient monitoring, teleconsultation, telemedicine

## Abstract

Background and objectives: The COVID-19 pandemic necessitated a shift in healthcare delivery, with both patients and physicians seeking alternatives to minimize hospital visits. Telemedicine has emerged as a viable solution, offering comparable health outcomes to conventional in-person consultations while improving patient satisfaction. This study aims to present our experience in establishing a teleconsultation service for antenatal patients, evaluating its impact on maternal and fetal outcomes, as well as patient satisfaction.

Methodology: A dedicated teleconsultation unit was initiated in April 2020 to manage antenatal care remotely. Patients received virtual consultations, with in-person appointments scheduled only when clinically necessary. Data were collected on teleconsultations, maternal and fetal outcomes, and patient satisfaction. Additionally, feedback from both patients and healthcare providers was recorded to assess the effectiveness of the service.

Results: Over one year, teleconsultation services were provided to 2,340 patients, including 1,550 antenatal cases and 790 with gynecological concerns. Among antenatal patients, 1,390 successfully completed teleconsultations, with 109 receiving teleconsultation services without prior appointments. Each antenatal patient, on average, had three virtual consultations and two in-person visits. A total of 76% of antenatal patients were scheduled for follow-up teleconsultations. Furthermore, 18% required in-person evaluations, 3% were directly advised of admission following teleconsultation, and 2% were referred for fetal medicine interventions, including chorionic villus sampling, amniocentesis, or advanced ultrasound assessments. The average duration of a teleconsultation was 5 minutes and 15 seconds, and 90% of patients reported satisfaction with the service.

Conclusion: Teleconsultation proved to be an effective strategy for reducing the need for physical visits while maintaining favorable maternal and fetal outcomes. High patient satisfaction rates further highlight its feasibility as a sustainable model for antenatal care.

## Introduction

Traditional obstetrics care revolves around obstetrics examination [1]. An obstetrician may feel incapacitated to provide healthcare if the examination is deferred. In this era of COVID-19, healthcare providers are in a dilemma to deliver health services [2]. Physicians and patients want to minimize hospital visits and find alternative ways of consultation. An obstetric patient may have concerns such as diet, supplements, minor symptoms like nausea or backache, interpretation of routine test reports, and birth preparedness, which can often be managed without a physical visit [3]. This is where telemedicine plays a role, referring to the provision of healthcare services by professionals using information and communication technologies to provide accurate medical information for diagnosis, treatment, and disease prevention, particularly when distance is a key challenge [4]. Research indicates that telemedicine delivers health outcomes comparable to traditional healthcare methods while also enhancing patient satisfaction and increasing engagement in their care management [5]. A tele-consultation center was started in our institute amid the COVID pandemic as a necessity due to lockdown, rather than a choice. This document aims to share our experience in establishing a new teleconsultation unit at a leading tertiary care center in North India. Additionally, to recommend practical guidance for obstetricians on offering teleconsultation services, including patient evaluation, management, and fostering a strong physician-patient relationship.

## Materials and methods

The purpose of starting teleconsultation at our center was to keep providing healthcare during the nationally mandated COVID lockdown. Teleconsultation services were started with this goal in mind, however, there were many challenges in initiating the services. Herein, we present an audit of the teleconsultation unit in the Gynecology outpatient department (OPD) at the All India Institute of Medical Sciences (AIIMS), New Delhi.

Type and setting of the teleconsultation unit

A teleconsultation unit was started with synchronous audio modality and an electronic data sharing facility. A centralized teleconsultation department was established in the institute to support the teleconsultation units of various departments, including ours. Our department was provided with three mobile phones, one for each unit. The smartphones had a provision to install a video calling App or an electronic exchange of reports. There was a departmental computer to keep call log data.

Patient identification and scheduling in obstetric teleconsultation

Most patients received information about the start of teleconsultation services by enquiring from patient enquiry areas, and subsequently booked their teleconsultation appointments. However, many obstetric patients were not following up. Most of them were high-risk pregnancies, as this is a referral center. The patient data were collected from previous research records. We had access to details and phone numbers of some antenatal patients who were registered with us. However, complete data for all obstetric patients were not available, as not all antenatal patients are registered at every visit. To address this limitation, we referred to our research records to retrieve their phone numbers. With the help of the central teleconsultation facility of the institute, messages were sent to their registered mobile numbers to book a teleconsultation with us. The days and timing of teleconsultation were the same as their in-person visit timings.

Utilization of guidelines to suit the purpose of obstetric care at our center

Teleconsultation guidelines provided by the Ministry of Health and Family Welfare and other international guidelines were reviewed to formulate a plan for teleconsultation. A meeting was held to design the implementation plan based on these guidelines.

Designated manpower

There was one nodal officer for teleconsultation from each unit of the department. They were in charge of the management of the teleconsultation list, data, and records. Four people from each unit were designated on their respective days to provide teleconsultation. This team consisted of a faculty member, a resident, an intern, and a data operator. Senior resident provided teleconsultation on the phone, intern helped in documentation of teleconsultations, and the data operator dealt with managing the calls, data sharing, and management. The faculty member supervised the overall smooth functioning of the consultations and decision-making in patient management.

Documentation of teleconsultation

Documentation is more straightforward in centers with complete electronic data management. However, our center uses paper documentation and record-keeping. OPD cards, which are used for in-person consultations, were utilized for the consults. Consent was taken from all the patients verbally. This consent was documented for the record. All the necessary information was documented on the card. This included details of the patient such as name, Unique Identification Number, consent for teleconsultation, date, time of start and end of teleconsult with duration, patient history, investigations as shared by phone, documentation of health counseling and education, treatment provided, full name, signature, as well as the designation of the person providing the teleconsultation. A picture of this card was shared with the patient to ensure they had a complete record of the consultation for their reference and benefit. All the cards were stored in files with the date of the teleconsultation. These cards were handed over once the patient arrived for in-person consultations.

Record maintenance of teleconsultation services

An Excel sheet (Microsoft Corp., Redmond, WA) was maintained to record data for all the patients. This data was compiled at the end of each month. A separate record was maintained for patients requiring close follow-up or in-person care, to be attended to once healthcare facilities reopened after the lockdown. These patients were subsequently followed up closely, either via teleconsultation or in person when feasible.

This exercise helped us improve our teleconsultation services in several ways. Firstly, systematic documentation ensured easy tracking of patients and timely follow-ups. Secondly, identifying those needing in-person care aided effective triaging and resource allocation. Thirdly, patient feedback helped in improving the overall quality and responsiveness of teleconsultation services. Patient feedback was obtained informally during follow-up consultations, and feedback from the doctors was collected following the teleconsultation.

## Results

Table 1 presents the teleconsultation data of one unit from the department. It also depicts the data of the obstetrics teleconsultation services provided by a unit of the department in a year. A total of 2,340 patients were provided teleconsultation between the months of April 2020 to May 2021. Out of this, 1,390 were antenatal patients. Most of the patients were high-risk pregnancies already booked with us. There were 160 unsuccessful teleconsultations, due to inability to contact on their registered phone numbers (wrong number/switched off phones/patient not available/not reachable/did not pick up). A total of 1,060 teleconsultation patients were advised further follow-up, 256 patients required an in-person visit for further evaluation and management, while 48 were advised follow-up for potential admission due to the need for closer observation or intervention. Additionally, 26 patients were recommended for specialized fetal medicine interventions, including advanced ultrasounds, chorionic villus sampling, amniocentesis, and cordocentesis, to assess and manage high-risk conditions. The duration of teleconsultation ranged from 2 minutes to 17 minutes. The average duration of teleconsultation was 5 minutes and 15 seconds.

**Table 1:** Data of one year of teleconsultation services in obstetrics Data of one year of teleconsultation services in obstetrics

Name of the Centre/Department	Department of Obstetrics (Unit X)
Total number of appointments	2340
Gynaecologic patients	790
Antenatal patients	1550
Total number of successful antenatal teleconsultations	1390
Teleconsultations provided after extracting contact details/ without appointment	109
Unsuccessful teleconsultations	160
Wrong number	34
Switched off phones	25
Patient not available	12
Not reachable	55
Did not pick up	35
Management of patients who attended the teleconsulations	
Recommended for further follow-up	1060
Recommended in-person visit	256
Recommended follow-up for admission	48
Adviced fetal medicine interventions (specialized USG/ Chorionic villi sampling/amniocentesis/cordocentesis)	26
Average duration of teleconsultation	5 minutes and 15 seconds

**Figure 1:**
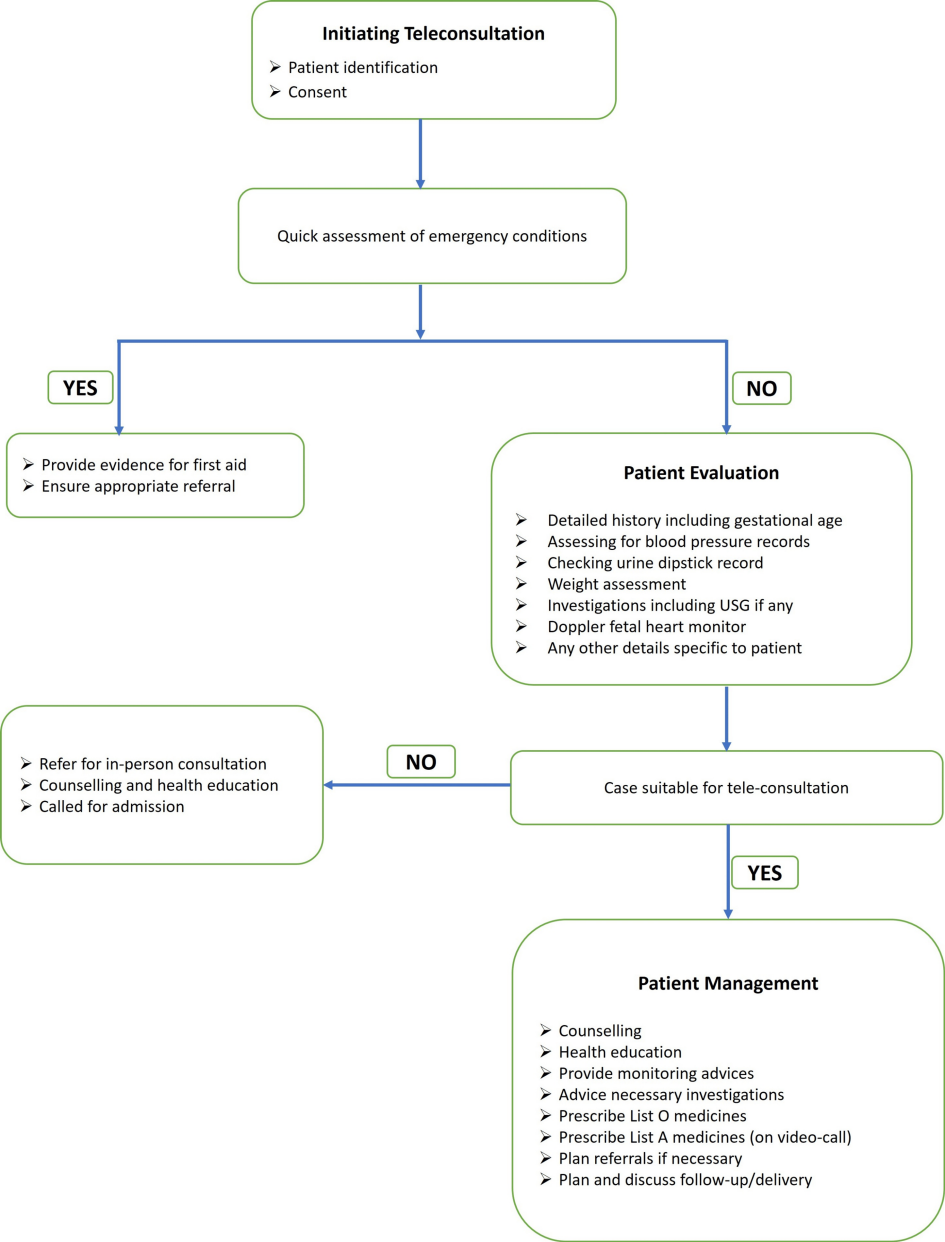
Flowchart for teleconsultation in obstetrics Flowchart for teleconsultation in obstetrics

Patient identification for teleconsultation was the first step. Teleconsultation was conducted only after the provider was reasonably certain of the patient's identity, ensuring both patient privacy and the delivery of appropriate treatment. By initiating the telemedicine consultation, the patient provides implicit consent; however, verbal consent was additionally recorded [4]. A very quick assessment for any emergency complaint was the next step. A review of the previous record of the obstetric patient was done, which was shared by the patient. After listening to the patient’s complaints or concerns, necessary information was sought. Once satisfied with the information needed, we proceeded with tele-management. The tele-management was either appropriate health education, counseling regarding the health condition, investigation needed, or treatment in the form of drugs or prescribing medications. A prescription issued through teleconsultation carries the same level of professional responsibility as one provided during an in-person consultation [4]. If follow-up was for continuation of care, the same medicines were prescribed, if necessary (List A). For common symptoms, over-the-counter medications were advised, as required (List O). Very few patients were prescribed as add-on medications (List B). A teleconsultation was provided as outlined in Figure 1.

**Figure 2:**
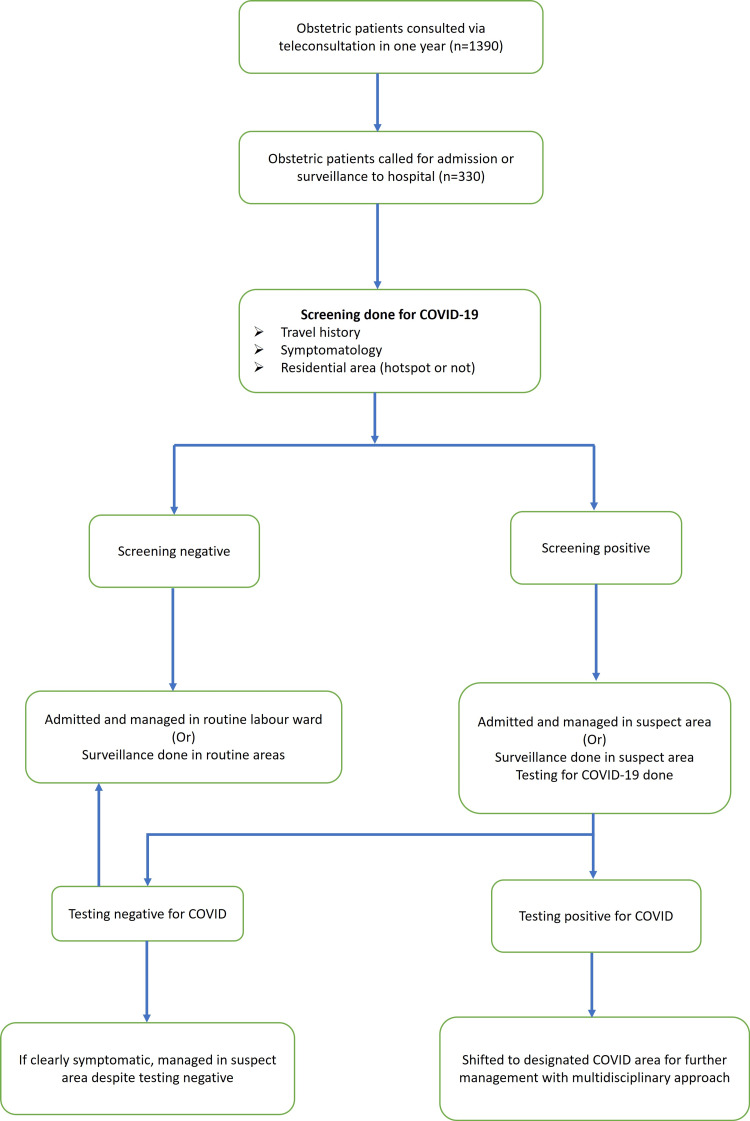
Flowchart for screening of Obstetric patients for COVID-19 Flowchart for screening of Obstetric patients for COVID-19

Out of 1,390 patients who were provided teleconsultation, 330 were called for either admission or maternal/fetal surveillance for various indications. They were all screened for symptoms or other high-risk factors for COVID-19. With the help of teleconsultations, the patients testing positive were first admitted to the suspect area. A test for COVID was done, and if found negative, they were managed in the routine labour ward unless the symptoms were really prominent. In that case, patients were managed in the suspect area, despite the testing being negative. Positive patients were managed in a specially designated COVID area with a multi-disciplinary approach (Figure 2).

Almost 90% patients appreciated the efforts. Many mentioned that it eased their anxiety and fears related to the disease amid the lockdown. Patient's feedback was obtained informally during follow-up consultations. More than 95% of the doctors providing teleconsultation were happy with the patient’s response during the feedback session. They felt that this eased both the doctors’ and the patients’ concerns.

## Discussion

Teleconsultations in obstetrics can be classified according to the mode of communication into video, audio, or text-based consultations [4]. The American College of Obstetricians and Gynecologists (ACOG) has classified telehealth modalities into synchronous (audio/video), asynchronous (sending medical imaging), remote patient monitoring (collecting personal health and medical data from an individual in one location and electronically transmits the data to a physician in a different location), and mHealth (self-managed patient care using mobile phone) [5]. We provided synchronous audio consultation in most cases.

Setting up a telemedicine facility requires necessary hardware, suitable software, a reliable and secure internet connection, and resource assessments to evaluate equipment readiness. Physicians should meet the safeguard criteria before delivering telehealth services, which include federal, state, and local regulatory laws and licensure requirements. The board of governors in the supersession of the Medical Council of India has given Telemedicine Practice Guidelines for any specialty [4]. When it comes to telemedicine in obstetrics, maintaining a record of patients in the form of e-files or paper records is vital, as the patients are typically under supervised follow-up. An obstetrician should not miss an important finding because a patient, who has shown a report from an earlier visit, does not find it necessary to mention it during a follow-up consultation.

Telemedicine can have an important role in routine antenatal care, in contrast to the general notion. Follow-up antenatal visits can be easily carried out by means of teleconsultation. A pregnant woman can transmit necessary information like weight, blood pressure, urine albumin, investigations, etc., electronically. Any new-onset symptom may be discussed. The need for an obstetric examination can be overcome by ultrasound examinations, which the patient might get done at nearby centers. It is not prudent to have an ultrasound examination on every visit; hence, well-spaced, necessary ultrasound scans should be advised as per patients’ needs. The WHO recommends a minimum of eight antenatal visits to ensure a positive pregnancy outcome and experience [6]. While obstetric examination cannot be replaced, ultrasound has become a practical alternative during the COVID-19 pandemic. WHO recommends that only one USG before 24 weeks is sufficient in low resource settings; however, it has been emphasized in recent analyses that more frequent ultrasounds may aid in strengthening referral systems in low resource settings [7]. Telemedicine in obstetrics can be used for genetic consultation and counseling, mental health check-ups, fetal echocardiography, monitoring of chronic medical disorders in pregnancy, review of non-stress tests, ultrasound evaluation by maternal fetal specialists, postnatal care, and monitoring [5, 8]. If a pregnant patient agrees to teleconsultation, apart from antenatal visits as a routine, she may be provided with some useful monitoring devices for personal use. A kit comprising a hand-held Doppler, fetal heart rate monitor, urine dipsticks, a blood pressure instrument, and printed manual instructions is invaluable in self-monitoring and reporting (Table 2)

**Table 2:** Home monitoring tools for patients under teleconsultation follow-up

Setting	Recommended Tools / Assessment Methods
Developed countries / Patients with access to resources	Blood pressure equipment
	Glucometer
	Hand-held doppler
	Fetal heart rate monitor
	Urine dipsticks
	Printed manual for instructions
Developing countries / Patients with limited resources	
Symptom-based assessment	Weight gain
	Tightness of jewelry like rings, anklets, and bangles
	Tightness of clothes on arms and legs
Additional assessment tools	Daily fetal movement count
	Blood pressure, sugars, and urine can be checked at a nearby center
	Instruction manuals can be provided

Incorporating telemedicine in healthcare decreases the number of unscheduled visits and is beneficial for both patients and healthcare providers. Patients may benefit financially by not having to travel long distances for consultations and minimizing time away from work and travel-related expenses. They can have support persons nearby during consultations, and unnecessary referrals or hospital visits can be avoided [9]. Opportunity for a subspecialist consultation, which otherwise might not be possible locally, is another advantage. Perinatal outcomes have been reportedly similar in patients managed with routine antenatal care and with incorporation of teleconsultation [10]. Improvements in obstetric outcomes, perinatal smoking cessation, improved breastfeeding rates, and schedule optimization for high-risk obstetrics have been noted with telemedicine facilities [11]. Healthcare providers may benefit due to less travel to outreach clinics, flexible hours, improved work-life balance, and potentially lower costs for clinic infrastructure [11, 12]. Additionally, we believe that an added advantage in the Indian context is the potential to reach women, especially from rural areas, who often avoid hospital visits as it affects their household chores. However, with the increasing prevalence of mobile phones in the general population, teleconsultation sessions can be facilitated, potentially leading to bookings at hospitals or clinics. This may also help promote institutional deliveries.

A unique advantage of establishing the teleconsultation unit was that we were able to conduct COVID-19 screening for all patients before calling them to our center for admission. Had these patients not been screened through teleconsultations, they would have presented directly to the emergency department, leaving no time for COVID-19 testing or receipt of results in emergency situations. This was to safeguard patient and healthcare provider interests. Table 3 shows the benefits of teleconsultation in obstetrics in the Indian scenario.

**Table 3:** Benefits of Teleconsultation in Obstetrics in Indian Scenario

Stakeholder	Benefit
Patients	Decreased number of unscheduled visits
	Not having to travel long distances for consultations
	Less missed work
	Financial benefits
	Presence of support persons close by
	Avoidance of unnecessary referrals
	Opportunity for subspecialist consultations
Obstetricians	Less travel to outreach clinics
	Flexible work hours
	Improved work-life balance
	Potentially lower costs for clinic infrastructure
	Fewer in-person interactions in a pandemic scenario
Healthcare services	Improved pregnancy booking rates
	Increased rates of institutional deliveries
	Improved ability to screen patients for COVID-19 before they present to the emergency department

There were several instances where in-person visits became necessary due to the diagnostic limitations of telemedicine. These included cases where patients presented with grossly abnormal investigation reports that required immediate clinical assessment, persistently high blood pressure indicative of potential hypertensive disorders, and alarming symptoms such as bleeding per vaginum or leaking per vaginum, which warranted urgent evaluation. Additionally, patients experiencing significant abdominal pain or exhibiting signs suggestive of early labor required timely in-person examinations and management. Such situations highlighted the critical need for clinical judgment in determining when teleconsultation alone was insufficient and an in-person visit was essential to ensure optimal maternal and fetal outcomes.

For a successful patient-physician relationship, communicating information effectively and compassionately is important. Use of synchronous modalities increases the trust between the two parties. Teleconferences should be performed in a quiet professional environment with a dedicated time specifically for video conferencing. The aim should be to offer the same level of care to patients receiving telemedicine as provided to patients in person. In our experience, teleconsultation helped us in terms of a successful patient-physician relationship. The patients and obstetricians were satisfied with the consults. It is advisable that the first consultation in obstetrics should not be a teleconsultation to build an adequate physician-patient trust and relationship. However, in case of necessity, like lockdown, curfews, and remote areas of living, a video consultation may be of help. The integration and advancement of information and communication technologies in healthcare delivery offer significant potential benefits for patients, providers, and payers in future health systems [13]. The potential benefits of telemedicine must be measured against the risks and challenges associated with its use, including the absence of the physical examination, variation in state practice and licensing regulations, and issues surrounding the establishment of the patient-physician relationship [14]. In the future, telemedicine may be a useful tool for diagnosis and clinical assistance of patients in remote areas of the world and/or rural areas far from a clinical center [15].

Limitations

There were many challenges while initiating teleconsultation services at our center. The antenatal patients, once booked with us, were not registered at every visit. This is done in order to avoid long waiting times and standing in line for prolonged hours for registration. Therefore, we did not have data on all obstetrics patients booked with us. To overcome this, we utilized our research data. Patients’ phone numbers were used, and a message was sent to these numbers asking them to book an appointment for teleconsultation with us. Another problem we faced was that many patients were not booking teleconsultations with us, despite getting the messages. With the phone numbers provided and retrieved by us, we directly called such patients on their clinic days. Thus, there were three categories of patients: (a) patients who took an appointment with us and called us on the day of the appointment, (b) patients who called us without any appointment, (c) patients whom we called for teleconsultation. We could provide our services to most of our booked patients with this approach. We also faced problems due to the non-availability of patient records/ e-files. For this, we asked the patients to share their previous records and investigations electronically. We maintained and stored this record. This record was utilized for repeat teleconsultations of the same patient.

## Conclusions

Teleconsultation has demonstrated its potential not only during the pandemic but also as a sustainable model for the future. Reducing unnecessary hospital visits and optimizing resource utilization, telemedicine can help decongest OPDs and ensure timely care, particularly for patients in remote and underserved areas. Beyond the pandemic, telemedicine can play a pivotal role in bridging the healthcare gap, improving access to specialized care, enhancing continuity of care, and supporting chronic disease management. With advancements in digital health technology and increasing acceptance among both providers and patients, telemedicine is poised to become an integral part of healthcare delivery systems, contributing to long-term improvements in health outcomes and healthcare equity.
